# Increased thrombospondin-1 levels contribute to epileptic susceptibility in neonatal hyperthermia without seizures via altered synaptogenesis

**DOI:** 10.1038/s41420-024-01837-3

**Published:** 2024-02-12

**Authors:** Yujie Zhai, Yao Cheng, Yi Yuan, Xianfeng Meng, Yang Li, Yan Wang, Tianpu Ren, Shucui Li, Hongliu Sun

**Affiliations:** School of Pharmaceutical Sciences, Binzhou Medical University, Yantai, 264003 China

**Keywords:** Epilepsy, Cellular neuroscience

## Abstract

Childhood febrile seizures (FS) represent one of the most common types of seizures and may lead to severe neurological damage and an increased risk of epilepsy. However, most children with fevers do not show clinical manifestations of convulsions, and the consequences of hyperthermia without seizures remain elusive. This study focused on hyperthermia not reaching the individual’s seizure threshold (sub-FS stimulus). Changes in thrombospondin-1 (TSP-1) levels, synapses, seizure susceptibility, and seizure severity in subsequent FS were investigated in rats exposed to sub-FS stimuli. Pharmacological and genetic interventions were used to explore the role of TSP-1 in sub-FS-induced effects. We found that after sub-FS stimuli, the levels of TSP-1 and synapses, especially excitatory synapses, were concomitantly increased, with increased epilepsy and FS susceptibility. Moreover, more severe neuronal damage was found in subsequent FS. These changes were temperature dependent. Reducing TSP-1 levels by genetic intervention or inhibiting the activation of transforming growth factor-β1 (TGF-β1) by Leu-Ser-Lys-Leu (LSKL) led to lower synapse/excitatory synapse levels, decreased epileptic susceptibility, and attenuated neuronal injury after FS stimuli. Our study confirmed that even without seizures, hyperthermia may promote synaptogenesis, increase epileptic and FS susceptibility, and lead to more severe neuronal damage by subsequent FS. Inhibition of the TSP-1/TGF-β1 pathway may be a new therapeutic target to prevent detrimental sub-FS sequelae.

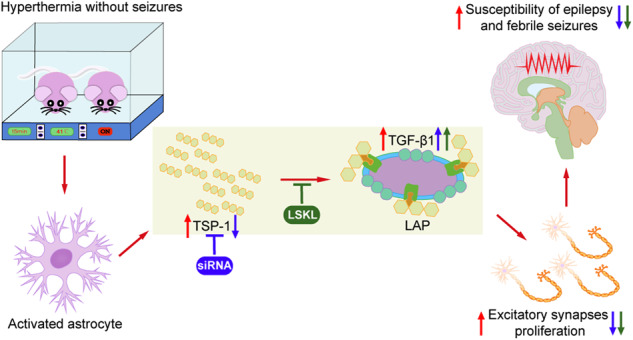

## Introduction

Febrile seizures (FS) mainly occur in children from 6 months to 5 years old [1]; approximately 2–5% of children worldwide experience FS [2]. A study of childhood FS found that children exhibited generalized seizures accompanied by fever >38°C (100 °F) without infection of the central nervous system [3]. Evidence suggests that FS may induce synaptic remodeling and increase the risk of neuronal injury-related diseases, such as epilepsy [4]. Children with a history of FS in infancy may develop temporal lobe epilepsy in adulthood [5]. Studies confirm that approximately 13% of epilepsy cases and 37.1% of children with sudden unexplained death are closely related to pediatric FS [6, 7]. Even after a simple FS lasting less than 15 min with no recurrence within 24 hours [2, 8], synaptic structures may be rebuilt [4], and the recurrence of FS is up to 50% in later life [9]. Additionally, epilepsy risk is significantly increased even in children with a minor FS [10, 11].

Hyperthermia-induced seizures are known to result in synaptic changes and an increase in the risk of epilepsy [12]. However, whether hyperthermia without seizures causes changes in epileptic susceptibility or seizure severity in later life remains unclear. After all, most children have high fever without clinical manifestations of convulsions. Therefore, our study aimed to assess the effects of hyperthermia not reaching the individual’s seizure threshold (sub-FS stimuli).

In early childhood, children with FS have an approximately five times higher incidence of subsequent seizures than those without [3]. Our previous study confirmed increased epileptic susceptibility due to sub-threshold pilocarpine doses [13]. If sub-FS stimuli similarly increase epileptic susceptibility, exploring the underlying mechanisms will be advantageous to prevent epilepsy.

Epilepsy is characterized by hyperexcitability [14], and epileptic susceptibility is closely related to neuronal excitability [15]. In these processes, synapses play important roles in the development and information transmission of complex neural networks [16]. Evidence suggests that thrombospondin-1 (TSP-1) plays a vital role in synaptogenesis [17–19]. TSP-1, an extracellular matrix molecule that is mainly secreted by activated astrocytes, contributes to synaptogenesis, axonal sprouting, the formation of normal synaptic ultrastructures, and even reshapes neural circuits in various neurological disorders [18, 20–22]. Moreover, synapses do not proliferate in the absence of TSP-1 [20]. TSP-1 promotes synaptogenesis by activating latent transforming growth factor-β1 (TGF-β1) in latency-associated peptide (LAP) [23, 24]. The Leu-Ser-Lys-Leu (LSKL) peptide sequence, located in the LAP of the TGF-β1–LAP complex, is essential in the activation of TGF-β1 [23, 25]. Exogenously administered LSKL can, through its interaction with TSP-1, competitively inhibit TGF-β1–TSP-1 binding, thereby inhibiting TGF-β1 activation [26, 27].

This study explored the changes and underlying mechanisms of epileptic susceptibility after hyperthermia without seizures; rat pups were treated with sub-FS thermal stimuli of different intensities to evaluate subsequent synaptic and susceptibility changes. To examine possible mechanisms of altered susceptibility, pharmacological and genetic interventions were used to modulate TSP-1.

## Results

### Sub-FS stimuli may increase FS and epileptic susceptibility and promote neuronal injury

**Fig. 1:**
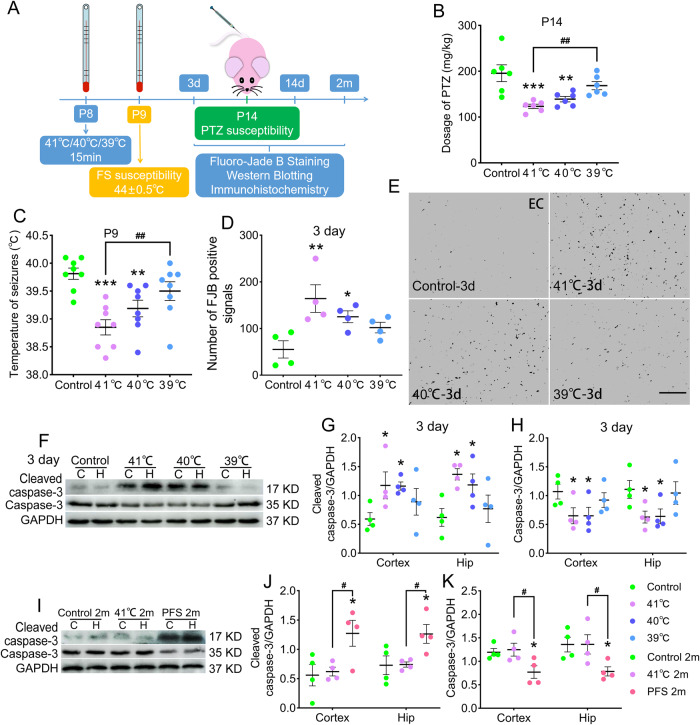
Sub-FS stimuli may increase FS and epileptic susceptibility and promote neuronal injury. **A** Experimental design. **B** Threshold dosages of PTZ at P14 (*n* = 6/group). **C** Threshold body temperature for induction of febrile seizures at P9 (*n* = 8/group). **D**, **E** Number of FJB-positive signals and representative images of FJB staining in the EC at 3 days (*n* = 4/group, scale bar = 50 μm). **F**–**K** Gray bands and normalized gray values of cleaved caspase-3 and caspase-3 relative to GAPDH at 3 days and 2 months (*n* = 4/group). Mean ± SEMs were presented. *P < 0.05, **P < 0.01, ***P < 0.001 vs control group and ^#^*P* < 0.05, ^##^*P* < 0.01 vs 41°C group (One-way ANOVA with LSD post-hoc test).

The body temperatures before and after modeling were measured. Significant increased body temperatures were found in sub-FS groups (Supplementary Fig. 1). FS and epileptic susceptibility were detected after 1 day (P9) and 6 days (P14) of sub-FS stimuli (Fig. 1A). After sub-FS stimuli, epileptic susceptibility was detected by injecting pentylenetetrazole (PTZ) via tail veins until seizures reached stage 4 or 5 by Racine’s criteria [28, 29]. Compared with that in the controls (sham treatment without sub-FS stimuli), the PTZ threshold doses were lower in the sub-FS groups (41 °C, *p* < 0.001; 40 °C, *p* = 0.002; Fig. 1B). Moreover, to detect FS susceptibility, P8 rats were administered sub-FS stimuli, then exposed to 44.0 ± 0.5 °C temperatures until seizures occurred on P9. The body temperature at which FS occurred was recorded. The threshold temperatures to induce FS in the 41 °C and 40 °C-exposed pups were lower than those in the control group (41 °C, *p* < 0.001; 40 °C, *p* = 0.004; Fig. 1C). The 39 °C treatment led to no significantly changes (Fig. 1B, C). Moreover, after 6 weeks of sub-FS stimuli, no significant differences between the 41 °C and control groups were found (Supplementary Fig. 2). The results suggest that increased epileptic susceptibility induced by neonatal sub-FS stimuli may be an early manifestation, and the impact of sub-FS stimuli may not persist into adulthood.

Neuronal damage was detected using fluoro-jade B (FJB) staining at 3 days. Sub-FS rat brains had increased numbers of FJB-positive signals (Fig. 1D, E). Additionally, we found elevated levels of cleaved caspase-3 (cortex: 41 °C, *p* = 0.038; 40 °C, *p* = 0.042; hippocampus: 41 °C, *p* = 0.012; 40 °C, *p* = 0.044; Fig. 1F, G), whereas those of caspase-3 were decreased (Fig. 1F, H) at 3 days after sub-FS stimulus. No significant changes were found in the 39°C group (Fig. 1F–H).

We also evaluated apoptosis at 2 months in the 41 °C sub-FS group. Compared to those in the prolonged febrile seizure (PFS) group, cleaved caspase-3 levels were dramatically lower with opposing changes in caspase-3 levels in the 41 °C group (Fig. 1I–K). No significant differences were found between the 41 °C and control groups (Fig. 1I–K). These results suggest that in the early stage, sub-FS may promote apoptosis, but these effects do not persist into adulthood.

### Sub-FS stimuli may aggravate neuronal damage and apoptosis induced by subsequent FS stimuli

**Fig. 2:**
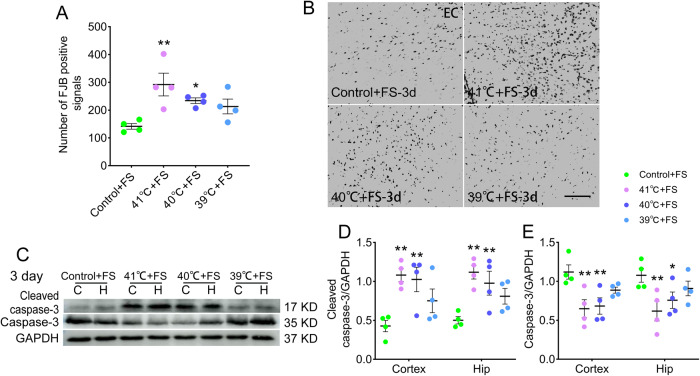
Sub-FS stimuli may aggravate neuronal damage and apoptosis caused by subsequent FS stimuli. **A**, **B** Number of FJB-positive signals and representative images of FJB staining in the EC at 3 days after FS stimuli (*n* = 4/group, scale bar = 50 μm). **C**–**E** Gray bands and normalized gray values of cleaved caspase-3 and caspase-3 relative to GAPDH (*n* = 4/group) at 3 days after FS stimuli. Mean ± SEMs were presented. *P < 0.05, **P < 0.01 vs control group (One-way ANOVA).

In those rat pups administered sub-FS treatment (41 °C and 40 °C) that subsequently experienced FS, neuronal injury was aggravated (3 days, 41 °C, *p* = 0.002; 40 °C, *p* = 0.031; Fig. 2A, B) detected by FJB staining. Synchronously, the cleaved caspase-3 levels were increased, whereas the caspase-3 levels were decreased (Fig. 2C–E), compared to those in the control group. These results indicate that sub-FS stimuli may increase neuronal injury and apoptosis in animals exposed to FS stimuli afterward.

### Sub-FS may increase TSP-1/TGF-β1, excitatory synapse, and glutamate levels

**Fig. 3:**
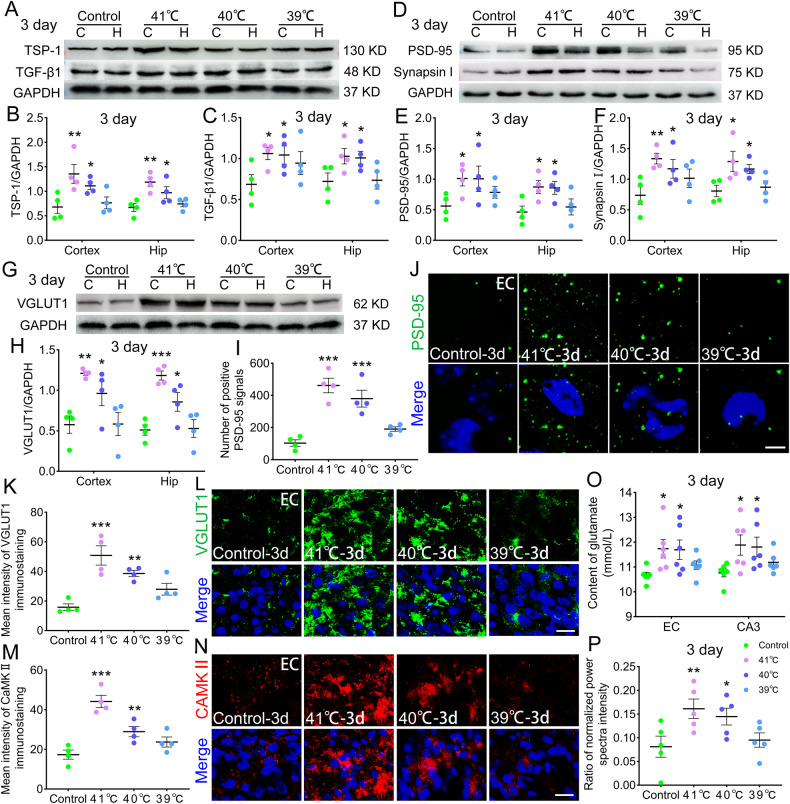
Sub-FS may increase TSP-1/TGF-β1, excitatory synapse and glutamate levels. **A**–**C** Gray bands and normalized gray values of TSP-1 and TGF-β1 relative to GAPDH at 3 days (n = 4/group). **D**–**H** Gray bands and normalized gray values of PSD-95, Synapsin I and VGLUT1 relative to GAPDH at 3 days (*n* = 4/group). **I**, **J** Number of positive PSD-95 signals and representative immunohistochemical results of PSD-95 (green) in EC at 3 days (scale bar = 5 μm, n = 4/group). Blue, DAPI. **K**–**N** Immunohistochemical results of VGLUT1 (green) and CAMKII (red) in EC at 3 days (scale bar = 10 μm, *n* = 4/group). Blue, DAPI. **O** Content of glutamate (*n* = 6/group) in EC and CA3 and (**P**) ratio of normalized power spectra intensity of beta waves (*n* = 5/group) at 3 days. Mean ± SEMs were presented. *P < 0.05, **P < 0.01, ***P < 0.001 vs control group (One-way ANOVA).

TSP-1 and TGF-β1 promote synaptogenesis in the brain [17]. The TSP-1 levels increased after sub-FS treatment (3 days, Fig. 3A, B; 14 days, Supplementary Fig. 3A, B). Likewise, the TGF-β1 levels were increased (Fig. 3A, C; Supplementary Fig. 3A, C).

We also detected the following multiple excitatory synaptic markers: postsynaptic density protein 95 (PSD-95), type I vesicular glutamate transporter (VGLUT1), calcium-calmodulin-dependent protein kinase II (CaMKII) [30–32], and synapsin I, a synaptic marker [33]. Western blotting results revealed increased levels of PSD-95 in the 41 °C and 40 C groups (e.g. 3 days: cortex: 41 °C, *p* = 0.04; 40°C, *p* = 0.043; hippocampus: 41 °C, *p* = 0.023; 40°C, *p* = 0.027; Fig. 3D, E; Supplementary Fig. 4A, B). Increases in VGLUT1, CaMKII, and synapsin I were similar to those in PSD-95 (Fig. 3D, F–H, K–N; Supplementary Fig. 4A, C-E). The increased PSD-95 levels were also confirmed by immunohistochemistry (Fig. 3I, J; Supplementary Fig. 4F, G). However, the levels of vesicular GABA transporter (vGAT), a marker of inhibitory synapses [34], were slightly decreased according to immunohistochemistry results (CA3: 41 °C, *p* = 0.028; 40°C, *p* = 0.047; Supplementary Fig. 5).

To evaluate the changes of excitement in the brains, we detected the concentration of glutamate, which mediates excitation transmission and hyperexcitation in the nervous system, by high-performance liquid chromatography (HPLC) at 3 days after sub-FS stimuli. Compared with those in the controls, the concentrations of glutamate in 41°C and 40°C sub-FS groups were increased in EC and CA3 (e.g., EC: 41°C, *p* = 0.017; 40°C, *p* = 0.019; Fig. 3O), in which excitatory synapse levels were significantly increased. Representative HPLC results of glutamate are presented in Supplementary Fig. 6.

Moreover, we recorded and analyzed electroencephalograms (EEGs) to evaluate electrophysiological activity in the brain at 3 days after sub-FS stimuli. When neuronal activity increases or is in an excitatory state, the beta power in the brain is increased [35]. Hence, we calculated the power spectral density (PSD) value in beta frequency bands. Results showed that compared with the control group, the power of beta waves was stronger in the sub-FS groups (41°C, *p* = 0.009; 40°C, *p* = 0.032; Fig. 3P). Representative EEG and PSD images are presented in Supplementary Fig. 7. The results suggest that when undergoing the sub-FS stimuli, the neuronal activity and excitability of the brain may be increased, accompanied with increased levels of excitatory synapse and glutamate.

### Reduced TSP-1 expression decreases susceptibility and neuronal injury induced by sub-FS stimuli

**Fig. 4:**
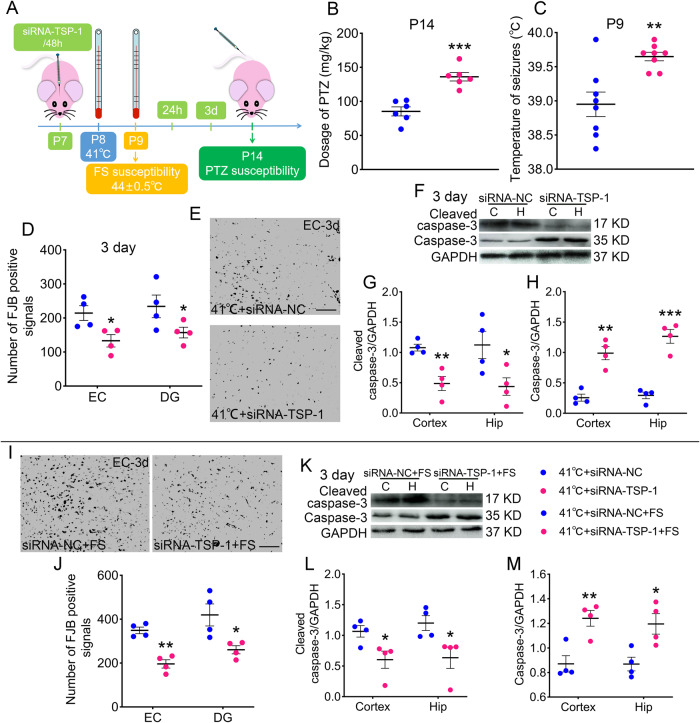
Reduced TSP-1 expression decreases susceptibility and neuronal injury induced by sub-FS stimuli and alleviates neuronal damage induced by subsequent FS stimuli. **A** Experimental design. **B** Threshold dosages of PTZ at P14 (*n* = 6/group). **C** Threshold body temperature for induction of febrile seizures at P9 (*n* = 8/group). **D**, **E** Analysis and representative images of FJB staining at 3 days after 41°C stimulus (scale bar = 50 μm). **F–H** Gray bands and normalized gray values of cleaved caspase-3 and caspase-3 relative to GAPDH at 3 days after 41°C stimulus. **I**, **J** Representative images (scale bar = 50 μm) and number of positive signals of FJB staining at 3 days after FS stimuli. **K**–**M** Gray bands and quantitative analysis of cleaved caspase-3 and caspase-3 at 3 days after FS stimuli. **D**–**M** n = 4/group. Mean ± SEMs were presented. *P < 0.05, **P < 0.01, ***P < 0.001 vs siRNA-NC group (Unpaired T-test).

FS and epileptic susceptibility were also assessed after small interfering RNA (siRNA) treatment (Fig. 4A). After the siRNA-induced reduction in TSP-1 expression, decreased epileptic susceptibility (*p* < 0.001; Fig. 4B) and increased threshold body temperatures of FS (*p* = 0.002; Fig. 4C) were detected.

The number of FJB-positive signals was markedly decreased in the siRNA-TSP-1 group compared to that in the siRNA-NC group (e.g., EC, *p* = 0.028; dentate gyrus [DG], *p* = 0.044; Fig. 4D, E). Moreover, in the siRNA-TSP-1 group, cleaved caspase-3 levels were decreased, whereas caspase-3 levels were increased (Fig. 4F–H).

### Reduced TSP-1 expression alleviates neuronal damage and apoptosis induced by subsequent FS stimuli

Neuronal damage was detected via FJB staining at 3 days after FS stimuli. The aggravation of FS-induced neuronal injury due to previous sub-FS stimuli was partly alleviated by siRNA transfection targeting TSP-1 (e.g., EC, *p* = 0.001; DG, *p* = 0.045; Fig. 4I, J). Furthermore, cleaved caspase-3 levels were decreased and caspase-3 levels were increased (Fig. 4K–M) in the siRNA-TSP-1 + FS group.

### Reduced TSP-1 expression decreases excitatory synapse and glutamate levels

**Fig. 5:**
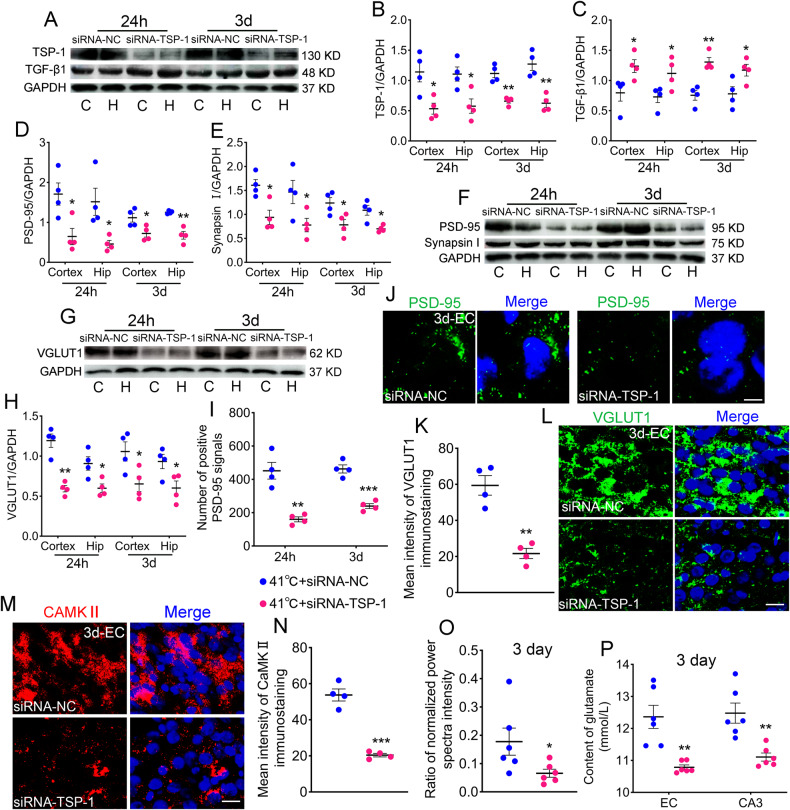
Reduced TSP-1 expression decreases excitatory synapse and glutamate levels. Gray bands and normalized gray values of TSP-1/TGF-β1 (**A**–**C**), PSD-95/synapsin I (**D**–**F**) and VGLUT1 (**G**, **H**) at 24 hours and 3 days (*n* = 4/group). The immunohistochemistry results of PSD-95 (green, scale bar = 3 μm, **I**, **J**), VGLUT1 (green, scale bar = 10 μm, **K**, **L**) and CAMKII (red, scale bar = 10 μm, **M**, **N**) in EC. **I**–**N** *n* = 4/group, Blue, DAPI. **O** Ratio of normalized power spectra intensity of beta waves (*n* = 5/group) and (**P**) glutamate content (*n* = 6/group). Mean ± SEMs were presented. *P < 0.05, **P < 0.01, ***P < 0.001 vs siRNA-NC group (Unpaired T-test).

TSP-1 and TGF-β1 levels were determined after siRNA-TSP-1 transfection. As expected, TSP-1 levels were lower in the siRNA-TSP-1 group than those in the siRNA-NC group (24 hours: cortex, p = 0.018; hippocampus, *p* = 0.02; 3 days: cortex, *p* = 0.004; hippocampus, *p* = 0.002, respectively; Fig. 5A, B), whereas TGF-β1 levels were increased (Fig. 5A, C). Accompanying reduced TSP-1 expression, PSD-95 (e.g. 24 hours: cortex, *p* = 0.022; hippocampus, *p* = 0.024; Fig. 5D, F), synapsin I (Fig. 5E, F), and VGLUT1 (Fig. 5G, H) levels were also synchronously decreased in the siRNA-TSP-1 group. The immunohistochemistry findings regarding PSD-95 were consistent with Western blotting results (Fig. 5I, J; Supplementary Fig. 8A, B). Furthermore, the levels of VGLUT1 and CaMKII were decreased in immunohistochemistry (Fig. 5K–N). Moreover, at 3 days after 41°C stimuli, the concentration of glutamate was decreased with weaker PSD value in the beta frequency bands in the siRNA-TSP-1 group (Fig. 5O, P).

### LSKL treatment attenuates epileptic and FS susceptibility and prevents neuronal injury

**Fig. 6:**
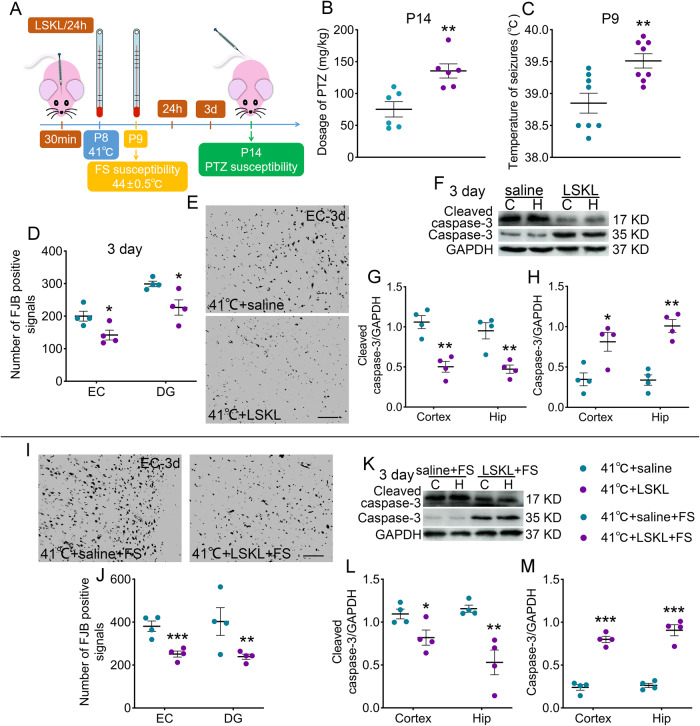
LSKL treatment decreases susceptibility and neuronal injury after sub-FS stimuli and alleviates neuronal damage induced by subsequent FS stimuli. **A** Experimental design chart. **B** Threshold dosages of PTZ at P14 (*n* = 6/group). **C** Threshold body temperature of febrile seizures at P9 (*n* = 8/group). **D**, **E** Number of positive signals and images of FJB staining (scale bar = 50 μm). **F**–**H** Gray bands and analysis of cleaved caspase-3 and caspase-3. **I**, **J** Images (scale bar = 50 μm) and number of positive signals of FJB staining at 3 days after FS stimuli. **K**–**M** Gray bands and analysis of cleaved caspase-3 and caspase-3 at 3 days after FS stimuli. **D**–**M** *n* = 4/group. Mean ± SEMs were presented. *P < 0.05, **P < 0.01, ***P < 0.001 vs saline group (Unpaired T-test).

LSKL has been demonstrated as essential in the activation of TGF-β1 [25], and exogenously administered LSKL can competitively inhibit TGF-β1–TSP-1 binding, thereby inhibiting TGF-β1 activity [26]. Epileptic and FS susceptibility were assessed after LSKL treatment (Fig. 6A). The results showed that epileptic susceptibility was lower in the 41°C + LSKL group than that in the 41°C+saline group (*p* = 0.004; Fig. 6B). Moreover, the FS body temperature threshold was increased after LSKL treatment (*p* = 0.004; Fig. 6C), and the number of FJB-positive signals was significantly reduced following LSKL treatment (EC, *p* = 0.036; DG, *p* = 0.027; Fig. 6D, E).

Additionally, LSKL administration resulted in decreased cleaved caspase-3 levels (3 days: cortex, *p* = 0.002; hippocampus, *p* = 0.005; Fig. 6F, G) and increased caspase-3 levels (Fig. 6F, H).

### LSKL administration alleviates neuronal damage and apoptosis induced by subsequent FS stimuli

After LSKL administration (41°C + LSKL + FS group), the number of FJB-positive signals was reduced (EC, *p* = 0.004; DG, *p* = 0.046; Fig. 6I, J), with decreased cleaved caspase-3 (3 days: cortex, *p* = 0.039; hippocampus, *p* = 0.006; Fig. 6K, L) and increased caspase-3 (Fig. 6K, M).

### LSKL administration decreases excitatory synapse and glutamate levels

**Fig. 7:**
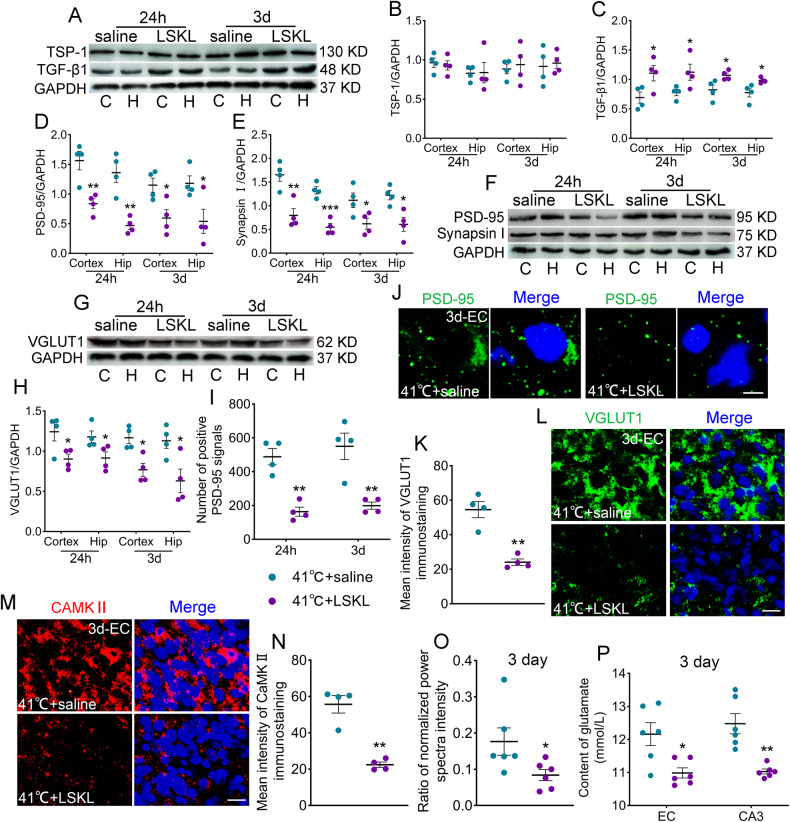
LSKL administration decreases excitatory synapse and glutamate levels. Gray bands and normalized gray values of TSP-1/TGF-β1 (**A**–**C**), PSD-95/synapsin I (**D**–**F**) and VGLUT1 (**G**, **H**) at 24 hours and 3 days (*n* = 4/group). **I**, **J** Number of positive PSD-95 signals and representative immunohistochemical results of PSD-95 (green) in EC at 3 days (scale bar = 3 μm, *n* = 4/group). **K**, **L** Immunohistochemistry results of VGLUT1 (green) in EC at 3 days (scale bar = 10 μm, *n* = 4/group). **M**, **N** Representative immunohistochemical results of CAMKII (red) in EC at 3 days (scale bar = 10 μm, *n* = 4/group). Blue, DAPI. **O** Ratio of normalized power spectra intensity of beta waves (*n* = 5/group) and (**P**) content of glutamate (*n* = 6/group) at 3 days. Mean ± SEMs were presented. *P < 0.05, **P < 0.01, ***P < 0.001 vs saline groups (Unpaired T-test).

TSP-1 and TGF-β1 levels were determined in LSKL-treated rats (41°C + LSKL group). Although the TSP-1 levels after LSKL treatment were not significantly changed compared to those of the saline group (Fig. 7A, B), the TGF-β1 levels were elevated by LSKL treatment similar to that observed in those after siRNA transfection (Fig. 7A, C). To verify synapse changes, PSD-95, VGLUT1, and synapsin I were detected at 24 hours and 3 days. The PSD-95 levels were considerably reduced by LSKL treatment (24 hours: cortex, *p* = 0.006; hippocampus, p = 0.002; 3 days: cortex, *p* = 0.024; hippocampus, *p* = 0.036, respectively; Fig. 7D, F) with downregulated VGLUT1 and synapsin I (Fig. 7E–H). The immunohistochemistry results confirmed the decreased PSD-95 levels in the 41°C + LSKL group (Fig. 7I, J; Supplementary Fig. 8C, D) with decreased levels of VGLUT1 and CaMKII (Fig. 7K–N). Moreover, the increased glutamate levels and the power of beta waves by sub-FS stimuli were partly reversed by LSKL intervention (Fig. 7O, P).

## Discussion

FS are induced by hyperthermia and represent one of the most common types of seizures [8]. FS can lead to irreversible synaptic reorganization [36] and recurrent spontaneous epileptic seizures [1]. However, most infants with hyperthermia do not experience seizures (sub-FS), and it remains elusive whether changes in epileptic susceptibility and synapses occur after sub-FS stimuli. Our findings indicate that sub-FS stimuli in rat pups may increase seizure and epileptic susceptibility and aggravates the neuronal damage caused by subsequent FS. Moreover, the synapse levels, especially excitatory synapse levels, were increased, with synchronously elevated TSP-1 and TGF-β1 levels. These changes correlated with temperature intensities. Decreases in TSP-1 expression by siRNA transfection or inhibition of TSP-1 activity by LSKL treatment significantly attenuated synaptogenesis and partly prevented the increased susceptibility.

Previous studies demonstrated that hyperthermia could change neuronal excitability. Hyperthermia led to epileptiform population spikes in hippocampal slices of immature rats [37] and reduced neuronal inhibition in the hippocampal CA1 subregion of neonate rats [38]. These findings suggest that hyperthermia may lead to absolutely or relatively increased excitability and influence seizure susceptibility. This suggestion is further supported by our results. Epileptic susceptibility was significantly increased in hyperthermia-treated pups, even without seizure induction. Pups treated with sub-FS stimuli were not only more likely to acquire FS, but also to sustain more severe FS-induced neuronal damage. However, there were no differences in epileptic susceptibility among groups at 6 weeks after sub-FS stimuli. Evidently, in those children experiencing hyperthermia without seizures, the susceptibility to epilepsy and FS may be elevated in the early period.

As the structural and functional basis of neural networks, synapses are vital in the development of the nervous system and the formation of hyperexcitatory diseases, including epilepsy [15, 16]. In the epileptic brain, neural network formation is characterized by abnormal synaptogenesis [15, 39], and remodeling of synapses can lead to increased excitability and epileptic susceptibility [40–42]. Based on our findings of increased susceptibility to both FS and epilepsy following sub-FS stimuli, we speculate that hyperthermia, even without reaching that of the seizure threshold, may induce synaptic changes in the brain. The increased levels of synapses, especially excitatory synapses, with elevated glutamate level were found after sub-FS stimuli. As the major excitatory neurotransmitter in the central nervous system, glutamate is mainly released from the presynaptic terminal via a calcium-dependent manner [43], and taken up by astrocytes and neurons [44]. Eventually, glutamate is packaged into glutamatergic neuronal vesicles, where glutamate is available for release [45]. Hence, the majority of glutamate is stored intracellularly. In order to detect the intracellular glutamate, we used liquid nitrogen to freeze and thaw the tissues repeatedly, and thoroughly crushed the cells with the ultrasonic crushing instrument. The results confirmed the increased concentration of glutamate, which indicated potentially elevated excitation neurotransmission after sub-FS treatment. Combined with the increased excitatory synapses and ratio of normalized PSD of beta waves after sub-FS stimuli, our results suggested that the increased excitability may be the mechanism underlying elevated susceptibility after sub-FS stimuli.

We observed the most substantial increases in the number of excitatory synapses in the EC and the CA3 region of the hippocampus, which are important in the formation of epilepsy and seizures. The hippocampus, one of the largest structures of the medial temporal lobe, is closely related to the occurrence of epilepsy [46]. Structural and functional defects of the hippocampus were found in patients with temporal lobe epilepsy, the most common type of epilepsy [47, 48]. During epilepsy formation, hyperexcitatory pathological changes occur in the hippocampus [46, 49]. For example, hippocampal mossy fiber sprouting constitutes excitatory feedback to the DG and CA3 region. This process provides the pivotal reorganization of excitatory connections [50, 51] and suppresses inhibitory transmission [52]. In the hippocampus, the predominant afferent pathway originates from the EC, the bridge between the hippocampus and cerebral cortex [53]. In general, information is transmitted from the EC to the DG through several tightly packed cellular layers, reaches the CA3 pyramidal cells, and is fed back to the EC via the CA1. CA3 axon collateralization plays an important role in this cycle [53]. Hence, it would be reasonable to speculate that increased excitatory synapses in the CA3 and EC induced by sub-FS stimuli may impact susceptibility and subsequent seizures. Our results support this hypothesis. Following sub-FS stimuli, the seizure threshold was significantly decreased, the number of excitatory synapses increased, and the neuronal damage was more severe in subsequent FS.

TSP-1, an astrocyte-secreted protein, is closely related to synaptogenesis in vitro and in vivo [54]. TSP-1 expression is essential for the ultrastructural formation of synapses, especially excitatory synapses [19, 55]. Increased TSP-1 levels promote synaptogenesis in young humans and newborn animals [56, 57]. Our previous study confirmed the contribution of TSP-1 in amygdala kindling-induced epileptogenesis by promoting synaptogenesis [17]. Reducing TSP-1 expression by siRNA transfection substantially inhibited synaptogenesis, especially excitatory synapse formation, and prevented epileptogenesis [17]. Interestingly, in sub-FS-treated rat pups, the levels of TSP-1 and synapse/excitatory synapses were concomitantly upregulated. Decreasing TSP-1 expression by siRNA transfection led to inhibited synaptogenesis (especially excitatory synapse formation), reversed seizure susceptibility, and attenuated neuronal damage in subsequent FS. Thus, TSP-1-induced synaptogenesis is critically involved in sub-FS-induced pathological processes.

TSP-1 affects synaptogenesis in the brain mainly by activating latent TGF-β1, and synapses deteriorate in the absence of TGF-β1 [17, 58, 59]. TGF-β1, a disulfide-linked homodimeric protein, is usually secreted in an inactive form bound to LAP for storage [60]. TSP-1 can activate TGF-β1 through K^412^RFK^415^, a specific amino acid sequence of TSP-1, interacting with the LSKL sequence of the latent TGF-β1–LAP complex [25]. Exogenous LSKL peptides competitively inhibit TSP-1 binding without affecting TSP-1 expression [23, 61, 62]. Following LSKL treatment, synaptogenesis and epileptogenesis were attenuated in amygdala-kindled animals [17]. Similarly, in sub-FS-treated pups, inhibiting TGF-β1 activation by LSKL treatment significantly reduced the number of synapses, especially excitatory synapses, prevented the increase of seizure susceptibility. Evidently, TSP-1 participated in the sub-FS-induced increase in susceptibility by promoting the formation of synapses/excitatory synapses, and the TSP-1/TGF-β1 pathway is the primary mechanism. Interestingly, the TGF-β1 levels were significantly increased after either LSKL administration or TSP-1-targeting siRNA transfection. The TGF-β1 upregulation may be compensatory owing to obstructed downstream functions.

Caspases are a family of cysteinyl aspartate-specific proteases that are highly conserved in multicellular organisms and function as central regulators of apoptosis and caspase-3 has been identified as a key mediator of apoptosis in neuronal cells [63]. Caspase-3 are also involved in non-apoptotic functions. For example, cleaved caspase-3 may induce local elimination of dendritic spines and spine loss without causing cell death and contribute to long-term depression [64, 65]. However, non-apoptotic effects of cleaved caspase-3 are varied and complex, even opposite. In prolonged seizures, neuronal G protein-activated inwardly-rectifying potassium (GIRK) channel subunits are cleaved by activated caspase-3, and down-regulation of GIRK channels may decrease their basal and GPCR-activated K^+^ current, disrupting the ability to dampen excitability [66]. Additionally, in hyperexcitatory diseases, such as epilepsy and febrile seizures, although synaptic pruning may occur, increased excitatory synaptic proliferation, and ultimately excitatory network formation, accompanied by apoptosis mediated by activated caspase-3, are the dominant pathological features [67, 68]. Hence, we speculate that during the increase of susceptibility induced by sub-FS stimuli, cleaved caspase-3 may contribute to the elimination of dendritic spines. However, the elimination effect of cleaved caspase-3 may be covered by the effects of TSP-1, which promotes axonal sprouting and synaptic proliferation [20, 22].

Notably, vGAT levels, a marker of inhibitory synapses [34], were slightly reduced in the hippocampal CA3, although the numbers of excitatory synapses were significantly elevated. We can speculate that the reduced number of inhibitory synapses aggravated the imbalance between excitation and inhibition in neuronal transmission pathways. In some pathological states, e.g., electrically-induced partial status epilepticus, directional pruning and clearing of inhibitory hippocampal synapses by activated microglia may contribute to the generation of hyperexcitable networks [69, 70]. Triggering receptor expressed on myeloid cells-2 (TREM2) is the pivotal molecule that mediates microglial synaptic clearance. Increased TREM2 levels promote microglial activation and greatly enhance synaptic phagocytosis [71, 72]. TREM2-regulated microglial phagocytosis may underlie the reduced number of hippocampal inhibitory synapses; further experiments are required to elucidate these contributions in detail.

Taken together, we found that even without seizures, hyperthermia may alter synaptogenesis, increase epilepsy and FS susceptibility in early periods, and lead to more severe damage by subsequent FS in a temperature-dependent manner. These changes are closely related to the TSP-1/TGF-β1 pathway. Our findings indicate that children experiencing hyperthermia without seizures may have an enhanced susceptibility in early periods and suggest the TSP-1/TGF-β1 pathway as a potential target to prevent epilepsy and FS.

## Materials and Methods

### Animals

Adult male and female Sprague-Dawley rats (280–300 g, Certificate No. SCXK 2019-0003; Jinan Pengyue Experimental Animal Center, Shandong, China) were given water and food *ad libitum*. After one week of adaptive feeding, male rats cohabited with female rats until pregnancy occurred. The birth date of the rat pups was recorded as P0. The experiments were performed between 9:00 and 17:00. A total of 308 rats were used in this study, of which 12 were excluded from analyses due to seizures. No rat died due to sub-FS stimuli. During the experiment, the examiners were blinded to data acquisition and analyses.

### Sub-FS stimuli and induction of prolonged febrile seizures

P8 rats were placed in a thermotank which was preheated for 2 h, while the temperature was controlled under the corresponding temperature conditions. The rats were randomly divided into control and three sub-FS groups (exposed to 39°C, 40°C, or 41°C [73] for 15 min). Rats experiencing seizure during the heating period were excluded from analyses. Control animals were placed in the unheated incubator.

To evaluate the injury in adulthood induced by juvenile sub-FS stimuli, PFS were induced on P9. Pups were randomly exposed to 44.0 ± 0.5°C temperatures for 90 min, and we ensured that the seizures lasted longer than 30 min [74]. The brains of rats from the PFS group were extracted at 2 months.

### Susceptibility assessment

First, epileptic susceptibility was detected by injecting PTZ via tail veins at P14 because rats at that age are old enough to stand and walk and presented obvious generalized seizures after PTZ treatment. PTZ (5 mg/ml, 0.05 ml/min; Sigma) was slowly injected into the tail veins of P14 rats until seizures reached stage 4 or 5 by Racine’s criteria[28, 29]. The threshold dose was calculated as follows: PTZ threshold dose (mg/kg) = (PTZ concentration (mg/ml) × infusion volume (ml))/ rat body weight (kg) [13].

Moreover, the threshold body temperature to induce seizures was measured to evaluate the susceptibility of the animal to FS. The threshold temperature for FS induction was determined as follows: P9 rats, which are susceptible to hyperthermia convulsions depending on the degree of brain development [75, 76], were placed in a thermotank heated to 44.0 ± 0.5°C [74] and retained in the tank until seizures were observed. The body temperature at which FS occurred was recorded.

### Western blotting

At 3 days, 2 weeks, and 2 months after sub-FS stimuli, rats were anesthetized and decapitated. Brains were quickly removed to separate the hippocampus and cortex on ice. After measuring protein content using a BCA Protein Assay Kit (P0012; Beyotime), proteins were separated using 10% and 12% SDS-PAGE gel (P0012AC; Beyotime) and transferred to a polyvinylidene fluoride membrane (220 mA, 60 or 100 min, respectively). Subsequently, these membranes were blocked with 5% skim milk for 3 h. After incubation with the primary antibodies anti-synapsin I (1:1 000; ab254349; Abcam), anti-PSD95 (1:1 000; ab2723; Abcam), anti-VGLUT1 (1:1 000; ab227805; Abcam), anti-TGF-β1 (1:1 000; ab179695; Abcam), anti-TSP-1 (1:1 000; A00667-1; Boster), anti-caspase-3 (1:1 000; 9662; Cell Signaling Technology), anti-cleaved caspase-3 (1:1 000; ab2302; Abcam), or glyceraldehyde-3-phosphate dehydrogenase (GAPDH; 1:1 000; AB-P-R-001; Goodhere) overnight at 4°C, and the horseradish peroxidase-conjugated secondary antibody (1:3 000; ZB-2301 and ZB-2305; Beijing Zhongshan) for 2 h at 37°C, the membranes were exposed (ImageQuant LAS 500, USA). Gel bands were analyzed using ImageJ (version 1.49; National Institutes of Health; United States) software and are presented as ratios to GAPDH.

### Immunohistochemistry

After adequate perfusion with 0.9% saline and 4% paraformaldehyde, extracted brains were dehydrated and embedded in Tissue-Tek, then sliced into sections. The brain sections were blocked with 10% bovine serum albumin and then incubated using the primary antibodies anti-PSD95 (1:200; ab18258; Abcam), anti-VGLUT1 (1:200; ab227805; Abcam), anti-CaMKII (phospho T286) (1:250; ab171095; Abcam), and anti-SLC32A1/vGAT (1:200; ab211534; Abcam) for 1.5 h, followed by incubation with the secondary antibody (fluorescein isothiocyanate-conjugated; 1:200; A0562 and A0568; Alexa Fluor 555-labeled Donkey Anti-Mouse IgG (H + L); 1:200; A0460; Beyotime) for 2 h in the dark at 37°C. After incubation with 4’,6-diamidino-2-phenylindole (DAPI; 1:3 000; 50 µl/section; C1005; Beyotime) at room temperature for 15 min in darkness, the slices were covered with coverslips and observed using a fluorescence microscope (Olympus, Japan). The obtained images were analyzed using ImageJ.

### Fluoro-Jade B staining

FJB staining was used to detect damaged neurons [77]. After successively being placed in 1% NaOH/80% alcohol for 5 min and 70% alcohol for 2 min, the slices were immersed in 0.06% potassium permanganate solution for 15 min. The slices were washed with water, then placed in 0.0004% FJB solution (AG310-30MG, EMD Millipore, USA) for 35 min in the dark, then rewashed with water and dried. After treatment with xylene for 3 min, the brain slices were observed under a fluorescence microscope (Olympus, Japan). The images were presented in grayscale and analyzed using ImageJ.

### LSKL and siRNA interventions

The most serious appearance after sub-FS stimuli was presented in the 41°C group. Therefore, 41°C treatment was used in the subsequent experiment to evaluate the effect of the intervention. The drugs were slowly injected into the lateral ventricle (3.5 mm caudal to and 1.5 mm left of bregma, 3 mm in depth) via a microinjection needle connected to a micro4 controller (World Precision Instruments, USA). The intracerebroventricular injection lasted for 2 min. After an additional 2 min, the needle was slowly removed.

To evaluate the effects of TSP-1, siRNA targeting TSP-1 [78] was synthesized by Tuoran Biological Technology (Shanghai, China) with the sequences: 5’-GCCAGUAUGUUUACAACGUdTdT-3’ and 5’-ACGUUGUAAACAUACUGGCdTdT-3’; negative control (siRNA-NC), 5’-UUCUCCGAACGUGUCACGUTT-3’, and 5’-ACGUGACACGUUCGGAGAATT-3’.

Twenty-four hours before sub-FS stimuli, the siRNA group was treated with anti-TSP-1 siRNA (0.2 μl, 0.5 μg/μl), whereas the control group was treated with negative siRNA (0.2 μl, 0.5 μg/μl). This intervention was performed every 48 h.

To suppress TGF-β1 activation by TSP-1, 30 min before sub-FS stimulus, LSKL (0.5 μl, 20 μg/μl) was injected into the lateral ventricle once every day [17] in the LSKL group, whereas saline (0.5 μl) was used in the control group.

### Glutamate concentration detection

After sub-FS stimuli, glutamate concentrations were detected by high-performance liquid chromatography (HPLC, Agilent Technologies, USA) equipped with a fluorescence detector [28]. After rat decapitation, the EC and CA3 were rapidly dissected on ice, mixed with 50% methyl alcohol (methyl alcohol: H_2_O = 1:1, 19 mL/g), then frozen and thawed four times with liquid nitrogen. After sufficient fragmentation of the cells and centrifuging (14 000 rpm, 4°C, 10 min), the supernates were diluted five times and filtered using a 0.45 and 0.22 μm microporous filter membrane. The standard curve was obtained (y = 531.69x - 17.623, R^2^ = 0.9999) with the glutamate standard (B21916, shyuanye, China). The diluent, AQC (A131410, Alladin, China) and sodium borate buffer (S885293, Macklin, China) were mixed in a ratio of 10:20:70, and underwent derivatization at 55°C for 10 min. After adding 400 μl of filtered water, we analyzed the samples using a Supersil ODS2 C18 column (200 mm × 4.6 mm, 5 µm, Elite Analytical Instrument Co., Ltd., China) at 30°C with 246 nm (excitation wavelength) and 399 nm (emission wavelength). The samples were separated by gradient eluent at a flow rate of 1 mL/min by the mobile phase which included eluent A (90 mM sodium acetate, 93% ultrapure and 7% acetonitrile, pH = 5.3) and eluent B (methanol:acetonitrile:water = 20:60:20). The gradient separation procedure was as follows: 0-5 min, 2% B; 6–10 min, 5% B; 11-12 min, 0% B; and 13–20 min, 100% B. The data were analyzed using Origin 9.0 and Prism 9.0.

### Electroencephalographic (EEG) testing

EEG was recorded after sub-FS. After rats were anesthetized and immobilized, the stainless steel electrodes (0.5 mm tips uncoated; A.M. Systems, USA) were implanted into the left cortex [anteroposterior (AP), -2.3 mm; mediolateral (ML), -2.1 mm; dorsoventral (DV), -1.3 mm] for EEG recording in accordance with brain atlases (the third section) [79]. Subsequently, when the pups woke, the EEG of each animal was continuously recorded for 3 h using a Powerlab device (1-80 Hz, AD Instruments, Australia). We calculated the PSD value in beta frequency bands. For normalizing the PSD value of EEG power, we analyzed the proportion of the beta frequency band in the five frequency bands (delta: 1-4 Hz; theta :4-8 Hz; alpha: 8-13 Hz; beta: 13-30 Hz; gamma: 30-80 Hz [80]) for each pup. The data were analyzed using Origin 9.0 and Prism 9.0.

### Statistical analyses

Rat numbers in each group were determined using the balanced one-way analysis of variance (ANOVA) based on our pre-experiment. In this study, all values are presented as mean ± standard error of the mean (SEM). All statistical analyses were performed with SPSS version 25.0 (SPSS Inc., USA). Statistical tests were justified, and the data met normal distribution and variance homogeneity. One-way ANOVA and an unpaired t-test were used to evaluate the differences between groups, the least significance difference (LSD) t-test was performed as a post-hoc test. In all analyses, *p* < 0.05 was considered to indicate statistical significance.

### Supplementary information

Original gel bands of Figure

Supplementary Figure 1-8

## Data Availability

The data and material in this study are available from the corresponding author on reasonable request.
