# Supplementary material for: Increased thrombospondin-1 levels contribute to epileptic susceptibility in neonatal hyperthermia without seizures via altered synaptogenesis
Source: Cell Death Discov. 2024 Feb 12;10:73. doi: 10.1038/s41420-024-01837-3 (PMC10861539; doi:10.1038/s41420-024-01837-3)

**Original gel bands of Figure 1**


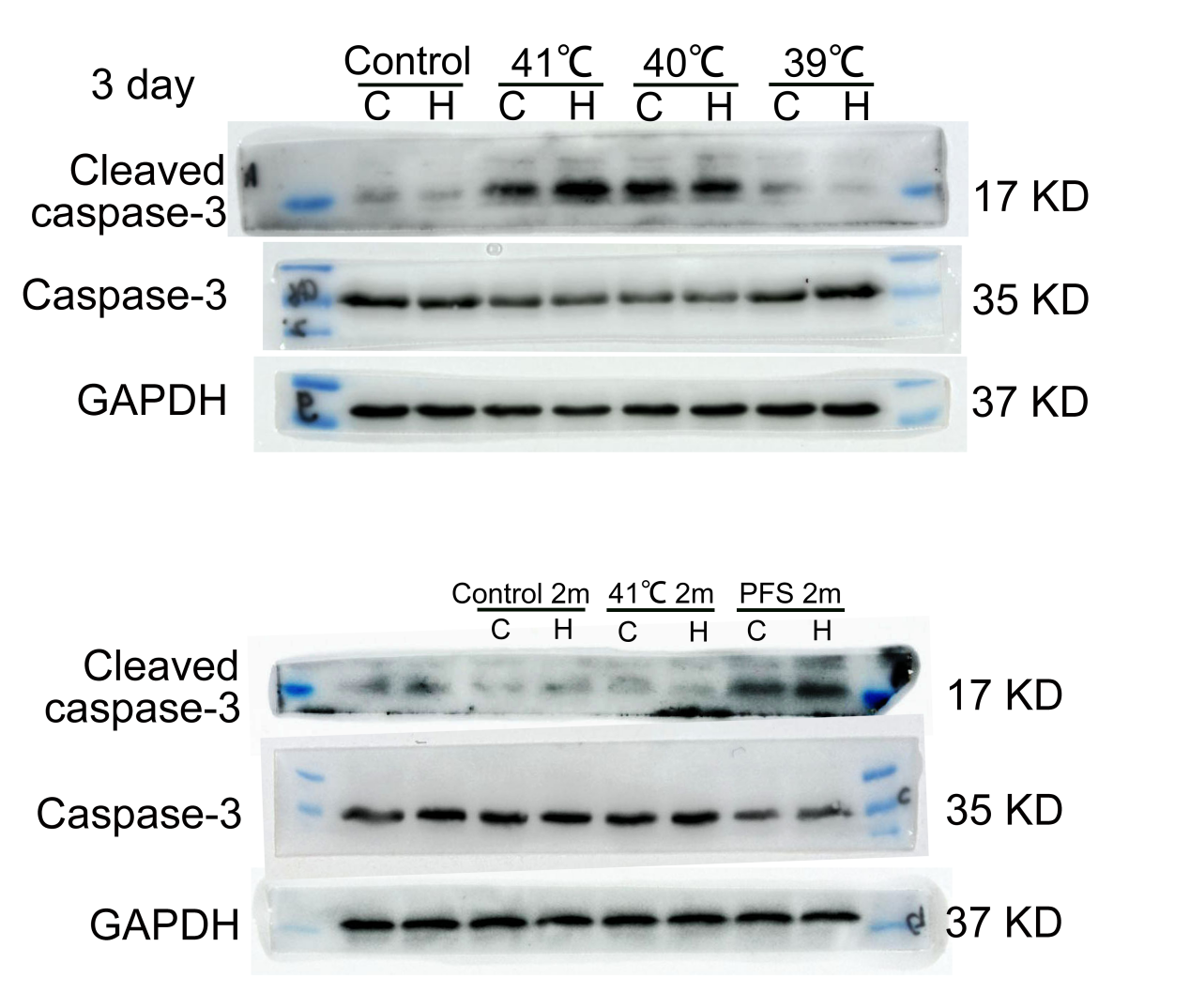


**Original gel bands of Figure 2**


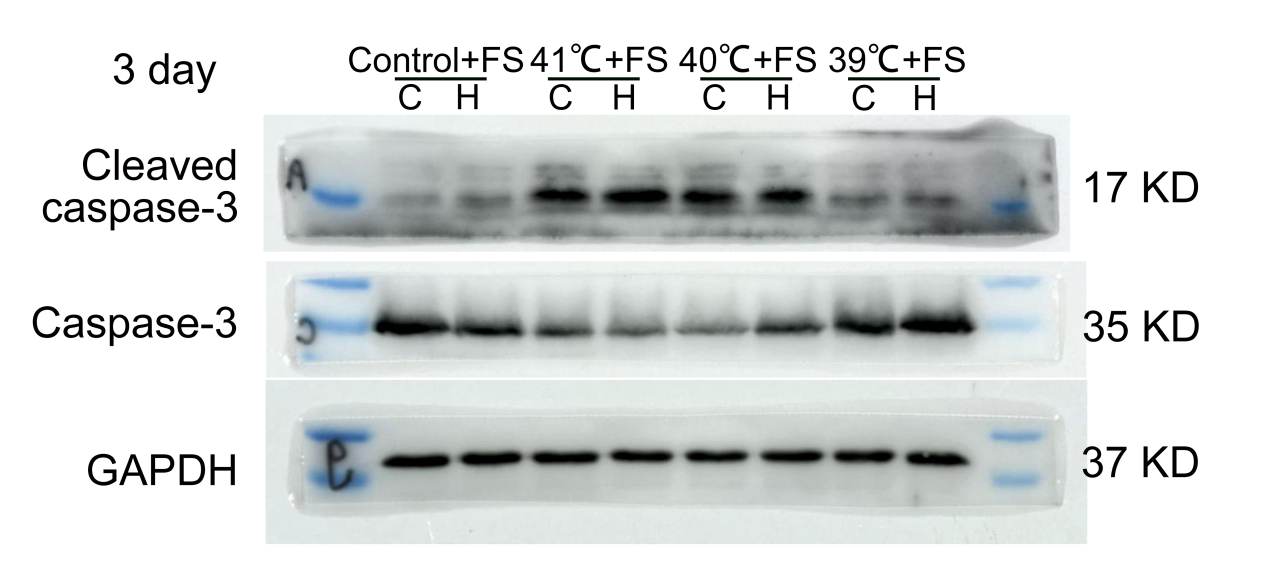


**Original gel bands of Figure 3**

**
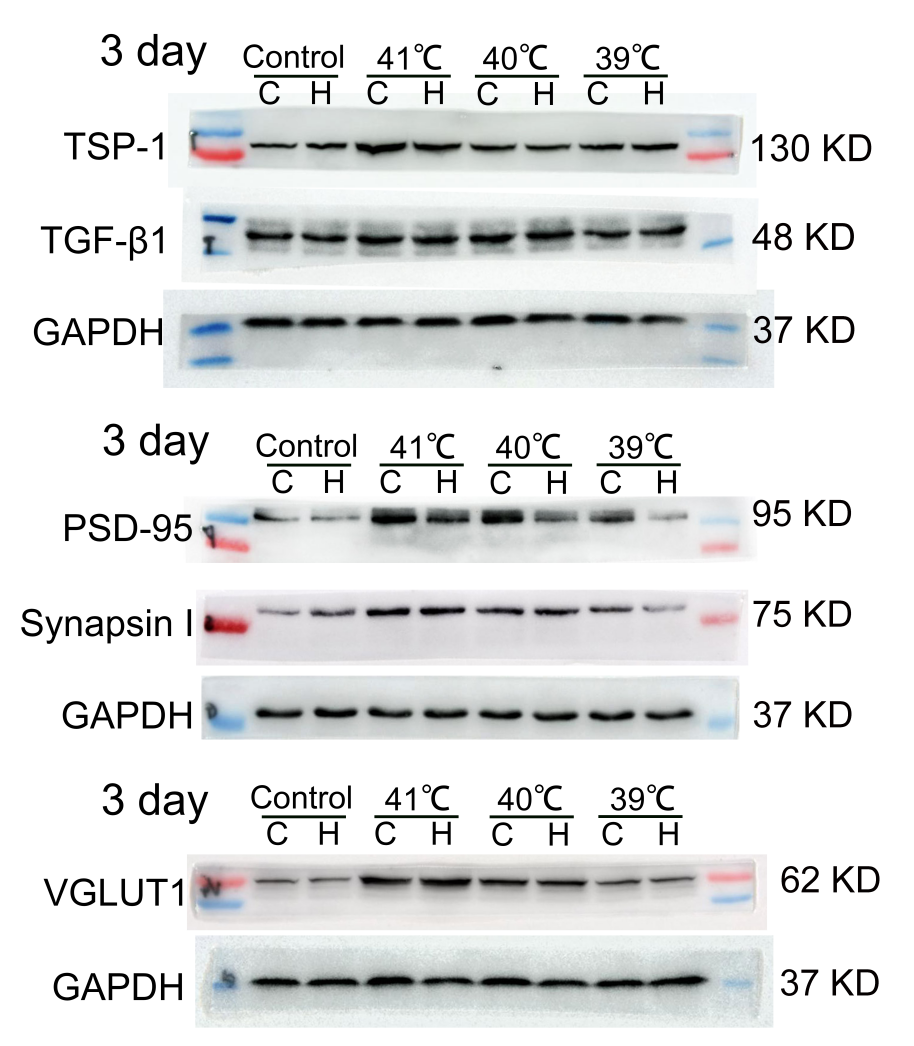
**

**Original gel bands of Figure 4**

**
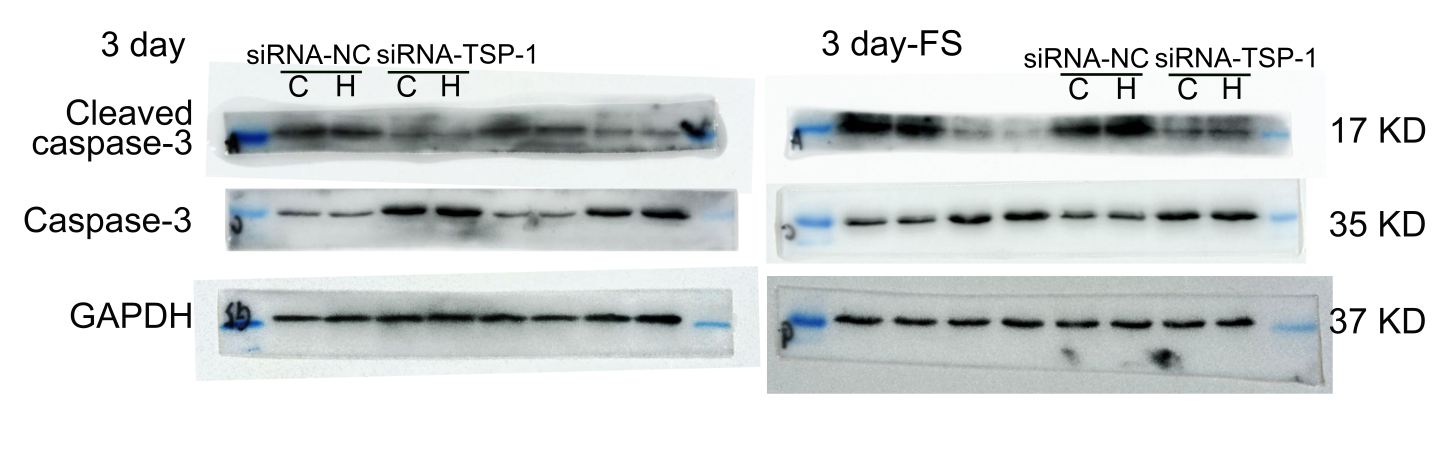
**

**Original gel bands of Figure 5**

**
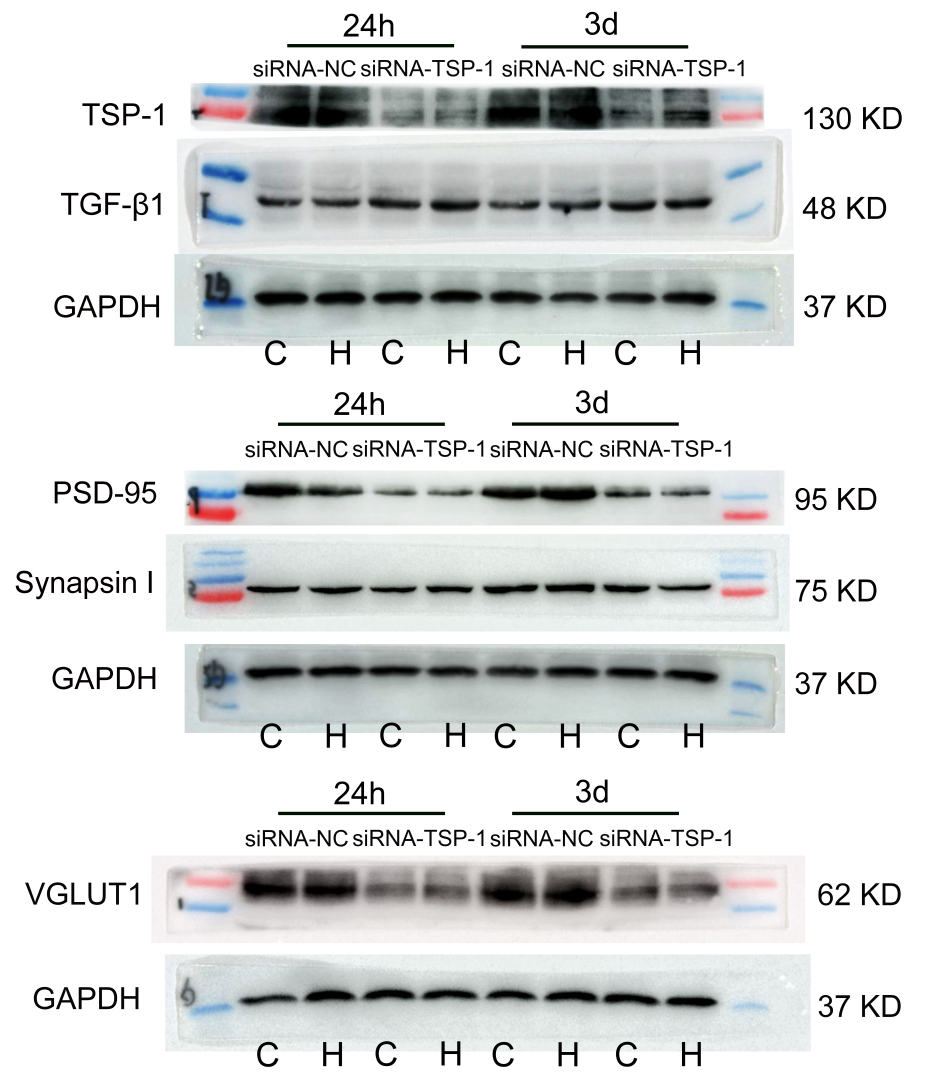
**

**Original gel bands of Figure 6**

**
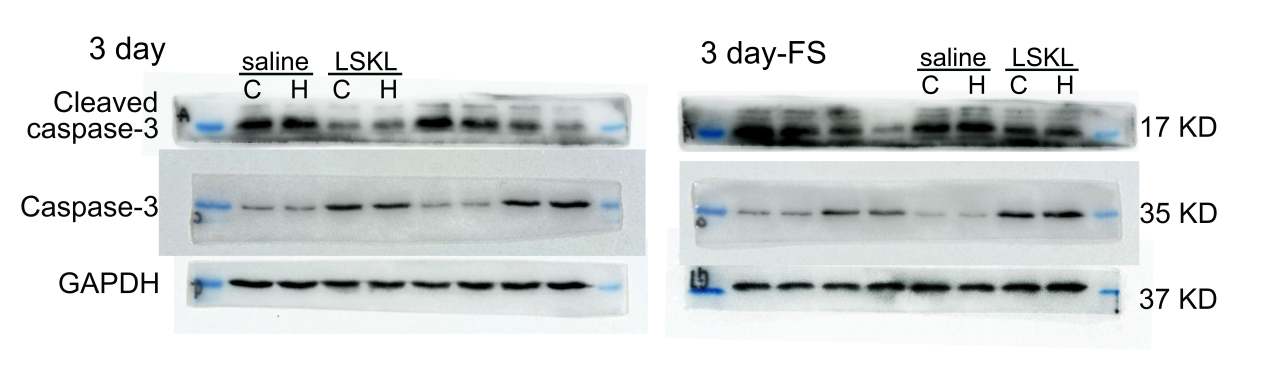
**

**Original gel bands of Figure 7**

**
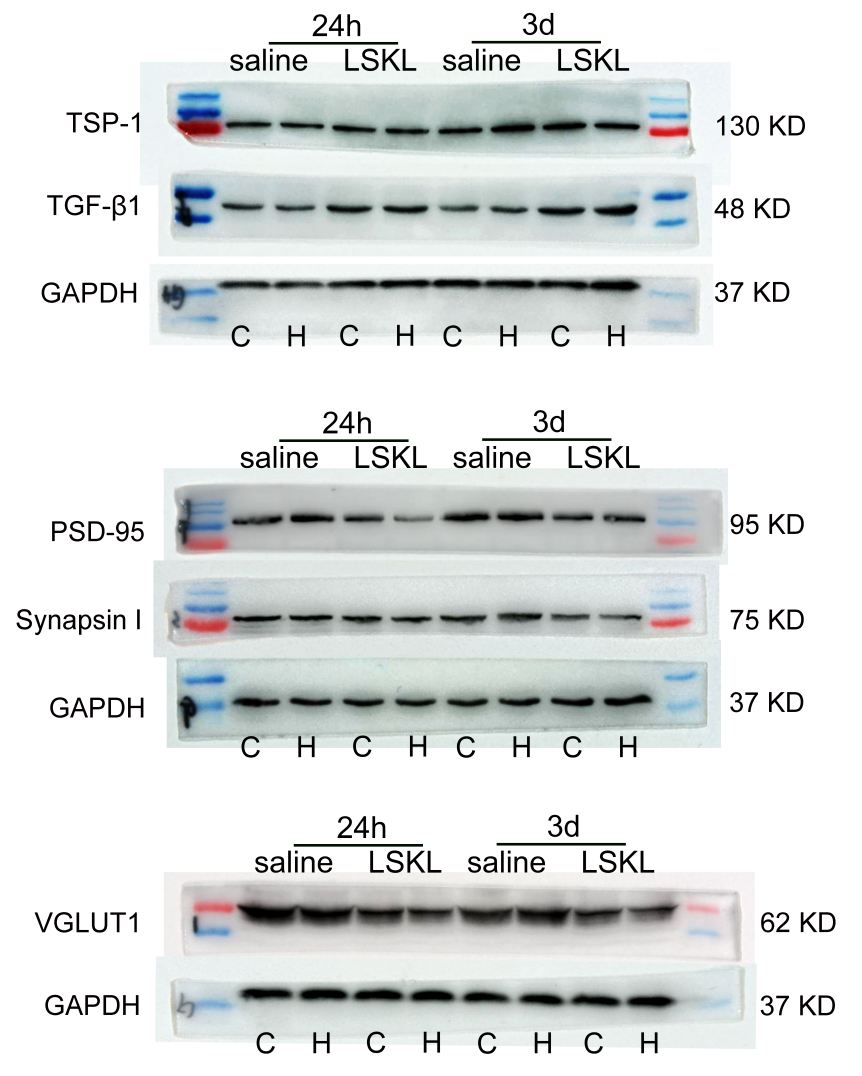
**

**Original gel bands of Supplementary Figure 3**

**
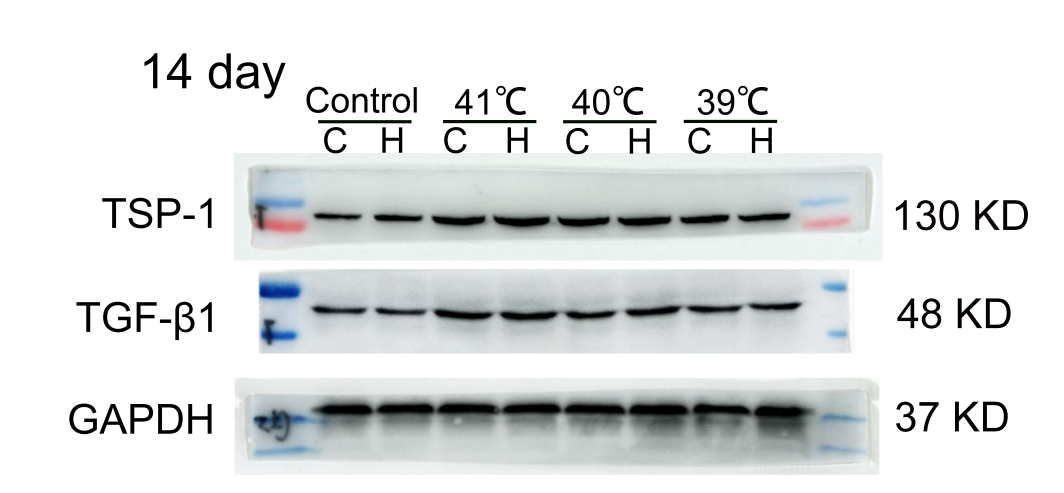
**

**Original gel bands of Supplementary Figure 4**


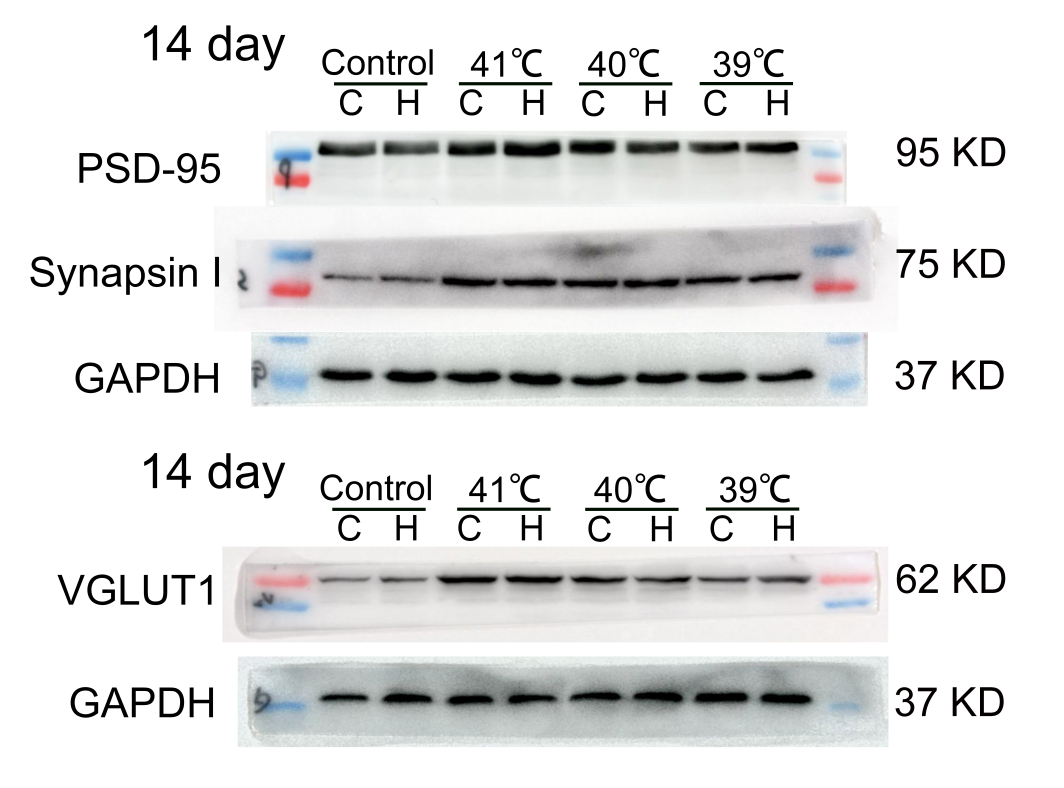

Supplement: Supplementary file 1 — Original gel bands of Figure [file 41420_2024_1837_MOESM1_ESM.docx]
