# Supplementary material for: Increased thrombospondin-1 levels contribute to epileptic susceptibility in neonatal hyperthermia without seizures via altered synaptogenesis
Source: Cell Death Discov. 2024 Feb 12;10:73. doi: 10.1038/s41420-024-01837-3 (PMC10861539; doi:10.1038/s41420-024-01837-3)
Supplement: Supplementary file 2 — Supplementary Figure 1-8 [file 41420_2024_1837_MOESM2_ESM.docx]

**
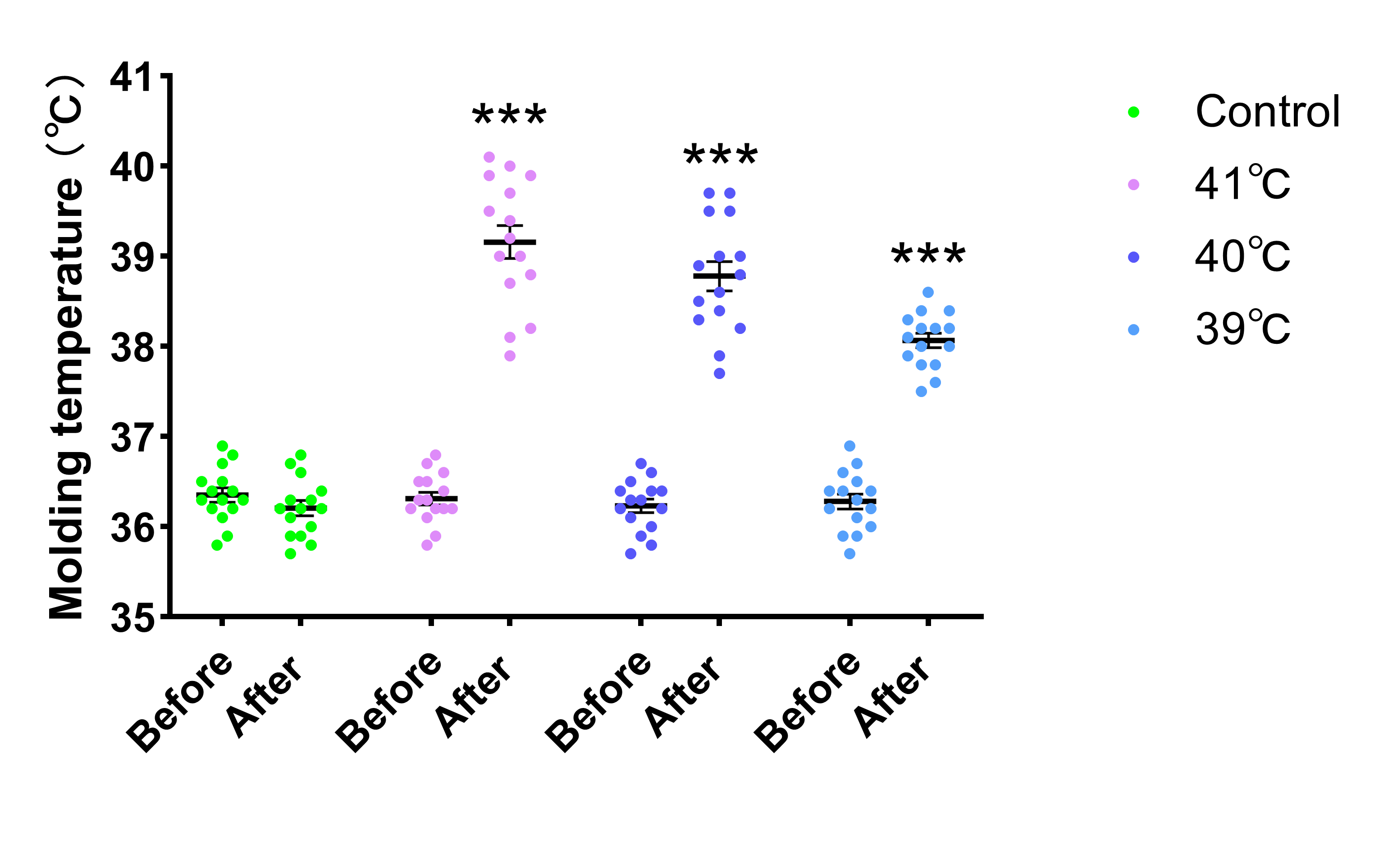
**

**Supplementary Fig. 1 The body temperature before and after modeling**

Increased body temperature was found in 41°C, 40°C and 39°C treated rats. n = 15/group. Mean ± SEMs were presented. ***P < 0.001 vs control group.


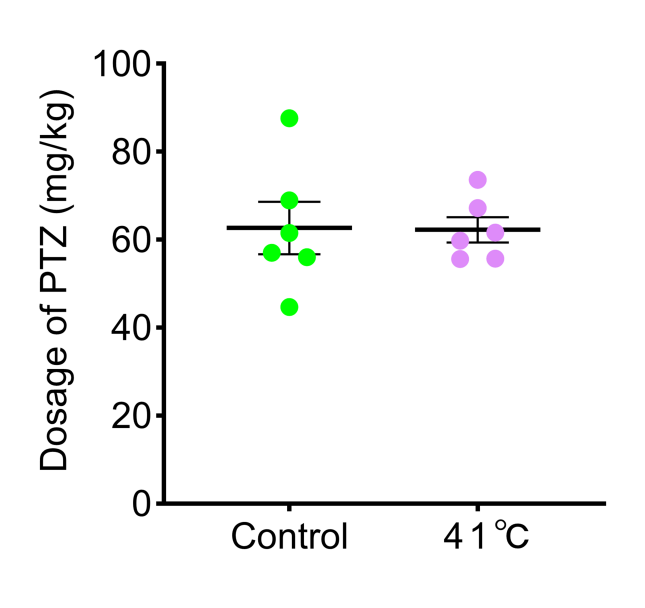


**Supplementary Fig. 2 Threshold dosages of PTZ at 6 weeks after sub-FS stimuli**

There was no difference in threshold dosage of PTZ between 41°C group and control group at 6 weeks after sub-FS stimuli. n = 6/group. Mean ± SEMs were presented.

**
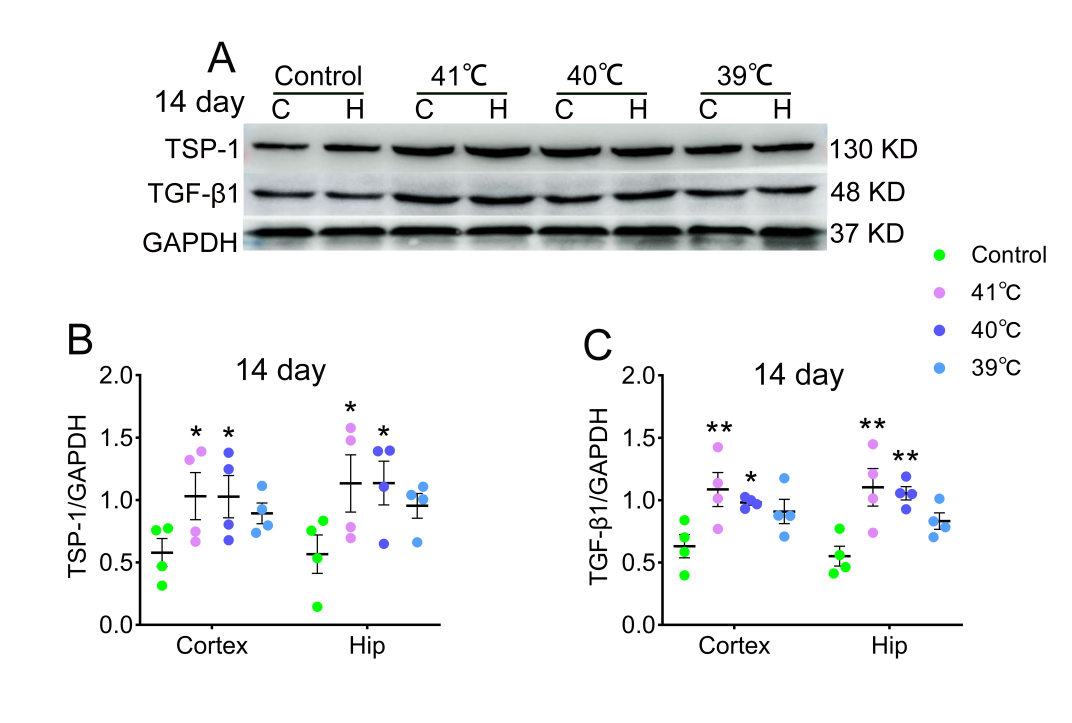
**

**Supplementary Fig. 3 The levels of TSP-1/TGF-β1 after sub-FS stimuli**

**A-C** Gray bands and normalized gray values of TSP-1/TGF-β1 at 14 days after sub-FS stimuli (n = 4/group). Mean ± SEMs were presented. *P < 0.05, **P < 0.01 vs control group.


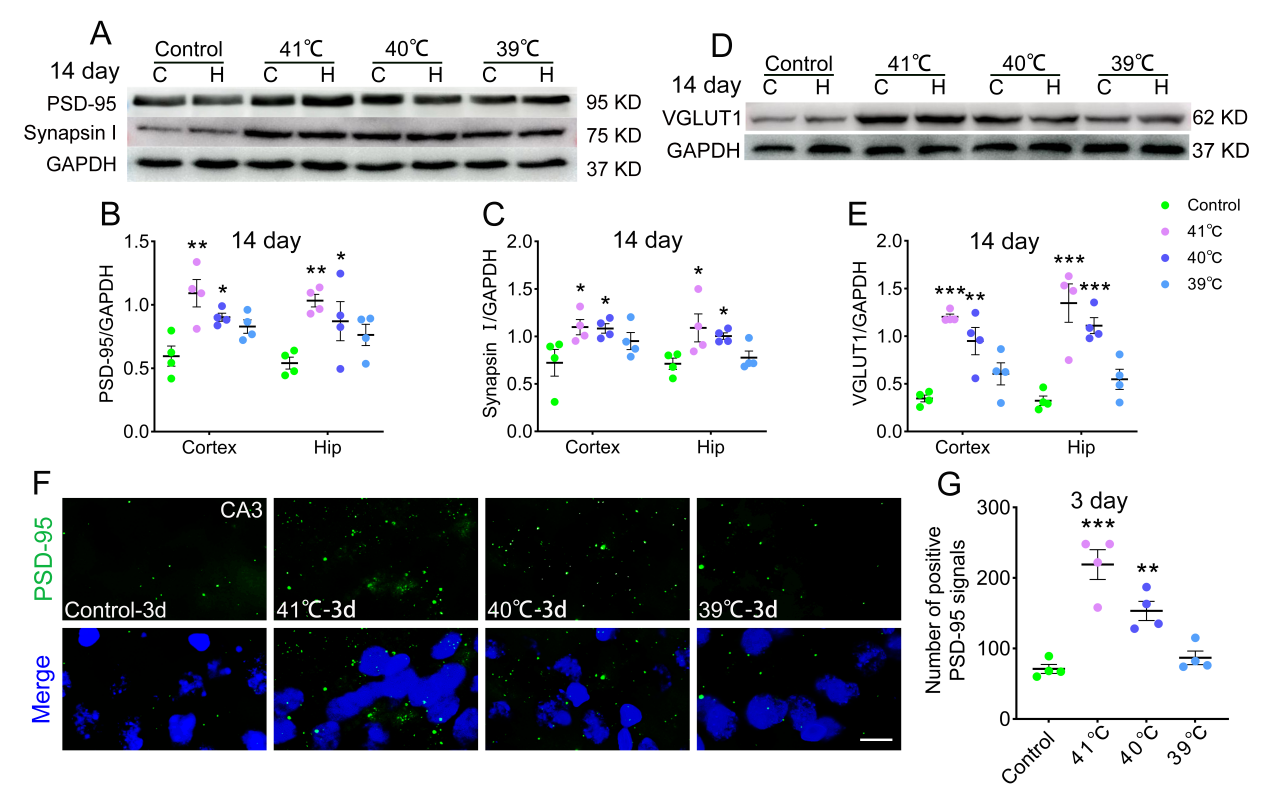


**Supplementary Fig. 4 The levels of excitatory synapse after sub-FS stimuli**

Gray bands and normalized gray values of PSD-95, synapsin Ⅰ (**A-C**) and VGLUT1 (**D, E**) at 14 days. **F, G** Immunohistochemical results of PSD-95 (green) in CA3 at 3 days. Blue, DAPI. Scale bar = 5 μm. **A-G** n = 4/group. Mean ± SEMs were presented.


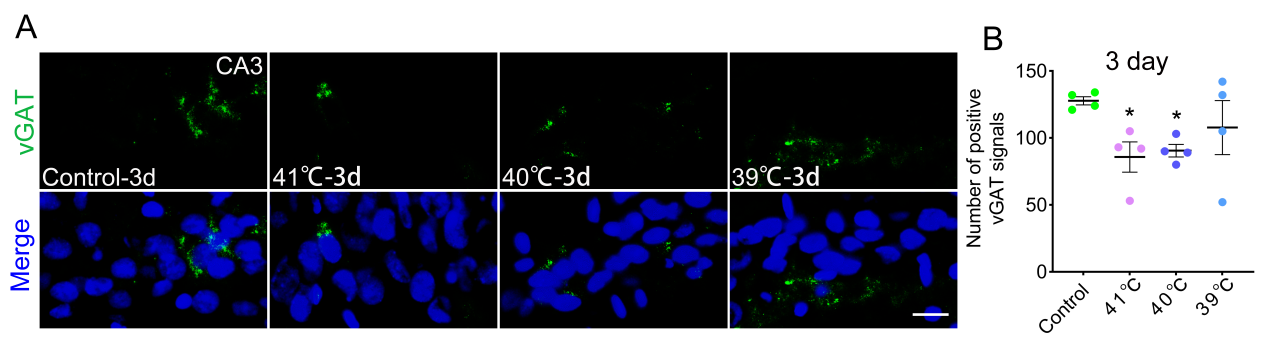


**Supplementary Fig. 5 Slightly reduced levels of inhibitory synapses after sub-FS stimuli**

**A, B** Representative immunohistochemical images (scale bar = 10 μm) and analysis of vGAT (green) in the CA3 at 3 days after sub-FS stimuli (n = 4/group). Blue, DAPI. Mean ± SEMs were presented. *P < 0.05 vs control group.


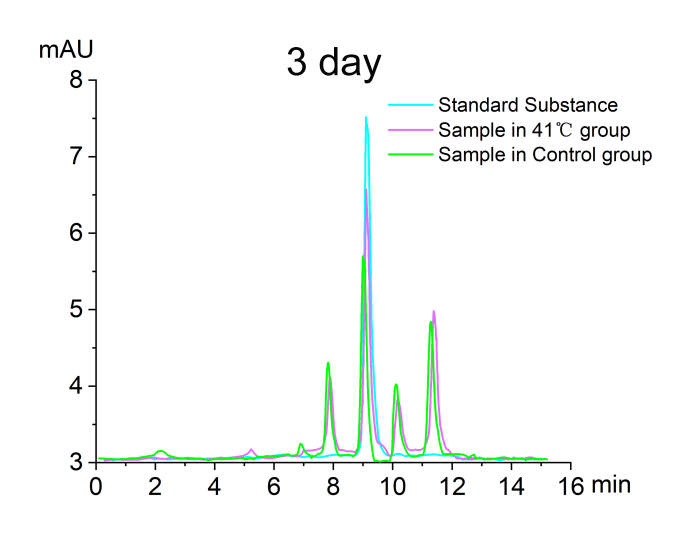


**Supplementary Fig. 6 Representative high-performance liquid chromatography results of glutamate levels**

Representative high-performance liquid chromatography results of glutamate levels in EC at 3 days after sub-FS stimuli.


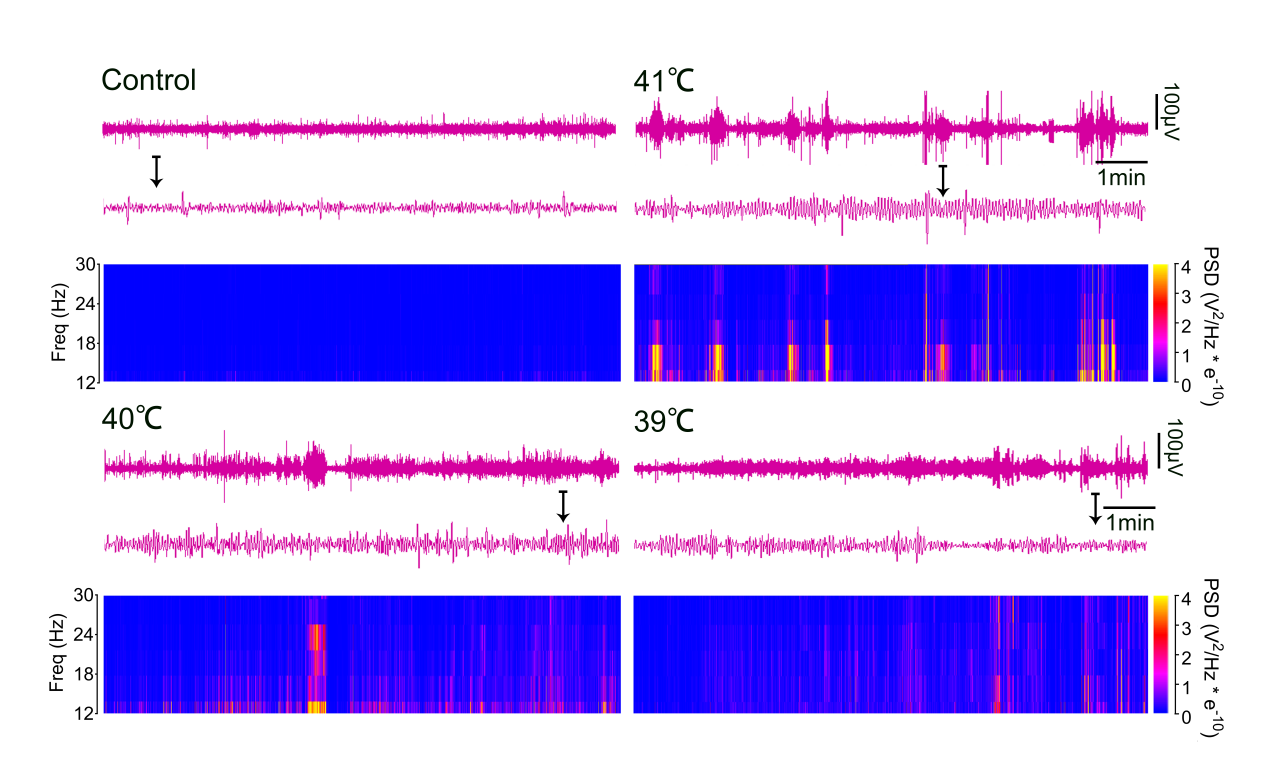


**Supplementary Fig. 7 Representative EEG and PSD analysis of beta waves**

Representative EEG and PSD analysis of beta waves between control and sub-FS groups at 3 days after sub-FS stimuli.


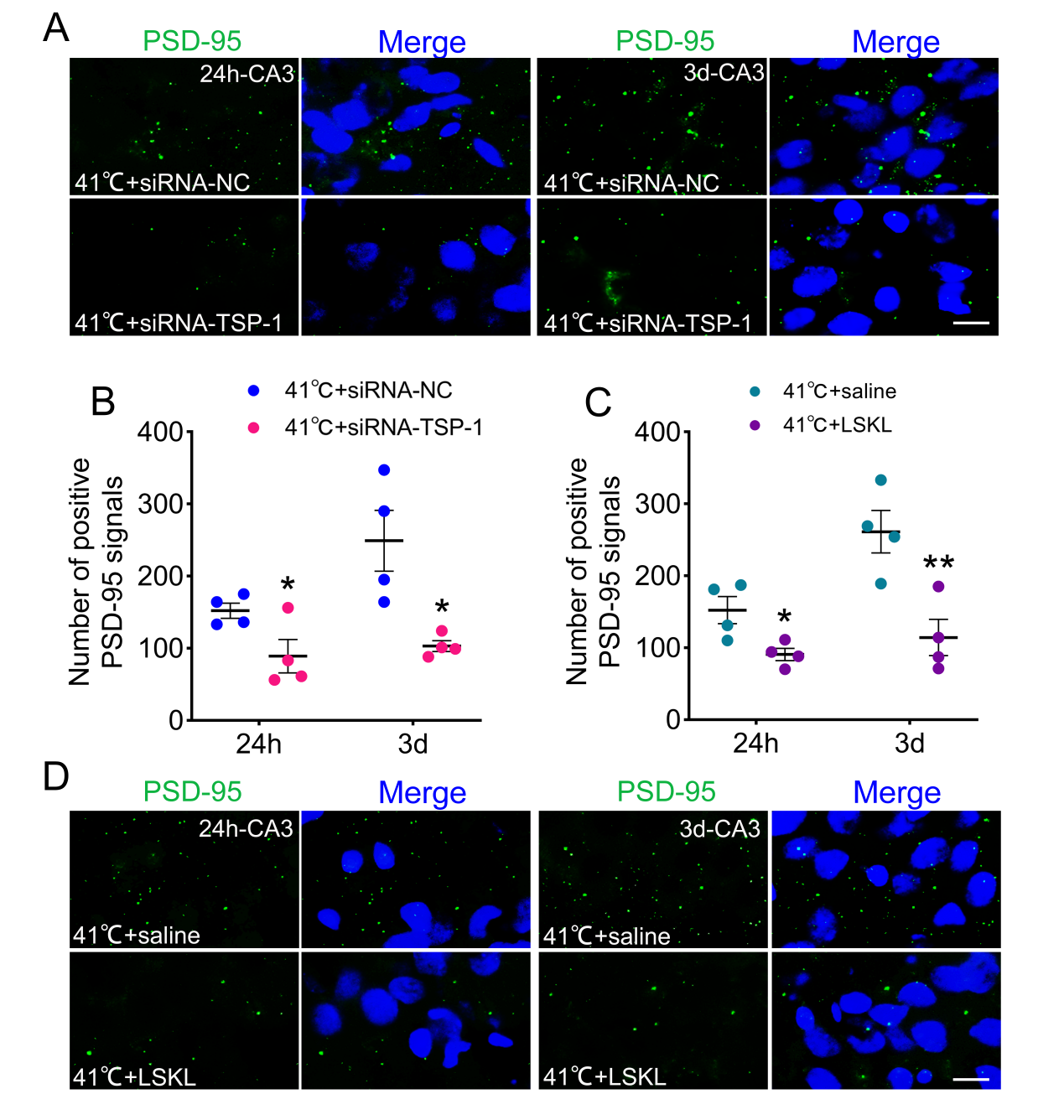


**Supplementary Fig. 8 siRNA and LSKL treatment decrease excitatory synapse levels after sub-FS stimuli**

The change of PSD-95 (green) in CA3 (scale bar = 5 μm) after siRNA intervention (**A, B**) and LSKL treatment (**C, D**). n = 4/group. Blue, DAPI. Mean ± SEMs were presented. *P < 0.05, **P < 0.01 vs control group.
